# Assessment of Case Complexity of Root Canal Treatments Using Contemporary Complexity Grading Systems: A Clinical Service Evaluation

**DOI:** 10.1111/iej.70039

**Published:** 2025-10-06

**Authors:** Nour Ghazi, Har‐amrit Singh, Edward Longbottom, Jeremy Hayes, Damian Farnell, Arindam Dutta

**Affiliations:** ^1^ School of Dentistry College of Biomedical and Lifesciences, Cardiff University Cardiff UK

**Keywords:** complexity, difficulty, endodontics, grading systems, root canal treatment

## Abstract

**Aim:**

To assess the complexity of root canal treatments allocated to Postgraduate Endodontology trainees at Cardiff University Dental Hospital (CUDH) using the English Clinical Standards for Restorative Dentistry (ECS) in comparison with the Dental Practicality Index (DPI), the EndoApp (EA), and the Endodontic Complexity Assessment Tool (E‐CAT).

**Material and Methods:**

Two‐hundred‐and‐one case records were evaluated by two calibrated examiners using each complexity assessment system. Inter‐examiner and intra‐examiner variability was calculated using Cohen's kappa coefficient. Statistical analyses compared the scores obtained for the same case using the different systems.

**Results:**

Most cases were assigned level 3 complexity using ECS, EA and E‐CAT (82%, 92% and 74.1%, respectively), and scores of 3–5 (78.6%) using DPI. EA consistently assigned higher complexity scores compared with ECS and E‐CAT. E‐CAT assigned lower complexity scores compared with ECS. A statistically significant moderate–substantial level of agreement was demonstrated between E‐CAT and ECS (weighted kappa = 0.647 [95% CI: 0.517 to 0.776], *p* < 0.001). A statistically significant fair level of agreement was demonstrated between EA and ECS (weighted kappa = 0.290 [95% CI: 0.113 to 0.466], *p* < 0.001) and EA and E‐CAT (weighted kappa: 0.385 [95% CI: 0.226 to 0.544], *p* < 0.001). A statistically significant weak positive correlation was found between DPI and ECS [Spearman's correlation coefficient (*r*_s_) = 0.202, *p* = 0.004], DPI and EA (*r*_s_ = 0.344, *p* < 0.001), and DPI and E‐CAT (*r*_s_ = 0.364, *p* < 0.001). The most common cause of increase in complexity scores was ‘canal negotiability’ for ECS (47%) and the ‘endodontic treatment need’ for DPI (84.1%). The unknown algorithm used by EA and E‐CAT prevented the identification of specific factors that contributed to the endodontic treatment complexity.

**Conclusion:**

The majority of cases treated at CUDH were of high complexity. E‐CAT assigned slightly lower complexity scores compared with ECS and EA, potentially due to its detailed assessment of factors. A weak positive correlation was found between the complexity grading systems. DPI's broader assessment justifies a cut‐off score of 3 for specialist referral due to the increased agreement with ECS, E‐CAT and EA at this threshold.

## 1 Introduction

In the United Kingdom, the provision of root canal treatment has increased significantly (NHS Business Services Authority 2024; Welsh Government 2024). Over the years, studies have shown that the success rate of root canal treatment (RCT) was high when appropriate techniques and procedures were applied (Ng, Mann, and Gulabivala 2008; Ng, Mann, Rahbaran, et al. 2008; Burns et al. 2022). While dentists have no control over the pre‐operative periapical status of the tooth, they are responsible for root canal preparation and filling procedures, which affect the treatment outcome. Inadequate restorative and endodontic treatment has contributed to an increase in the prevalence of apical periodontitis (Jakovljevic et al. 2020).

Identifying case complexity before treatment is vital to ensure patients are managed by appropriately skilled clinicians, which helps optimise the dental workforce (Rosenberg and Goodis 1992; Ree et al. 2003). Several grading systems have been developed to assess treatment difficulty, including the Restorative Index of Treatment Need (RIOTN) (Falcon et al. 2001), the American Association of Endodontics Case Difficulty Assessment (AAE) (American Association of Endodontists 2005), the Dutch Endodontic Treatment Index and Endodontic Treatment Classification form (Ree et al. 2003), and the Canadian Academy of Endodontics classification (Canadian Academy of Endodontics 1998). However, these systems face limitations such as poor to moderate reproducibility (Muthukrishnan et al. 2007) and limited practicality (Shah et al. 2020).

Hence, several other grading systems have been introduced recently to facilitate and standardise complexity assessment. These include the English Clinical Standards for Restorative Dentistry (ECS), the Dental Practicality Index (DPI), the Endo App (EA) and the Endodontic Complexity Assessment Tool (E‐CAT), the latter two tools utilising an electronic format for application.

ECS was proposed in 2019 (https://www.england.nhs.uk/publication/commissioning‐standard‐for‐restorative‐dentistry/). It standardises local commissioning of specialist and specialised services in restorative dentistry within the National Health Service in England. Long et al. (2022) compared three complexity grading systems (ECS, RIOTN and AAE) used in a university dental hospital setting and perceived the ECS to be more user‐friendly, quick to apply and simple as it did not use a complex points‐based system.

The DPI adopts a holistic approach by first considering the patient's medical, dental and social history (Dawood and Patel 2017). It also evaluates structural integrity, endodontic status and periodontal condition, with each domain scored as 0, 1, 2 or 6. The DPI score is the sum of these values. A total score of 1–2 suggests simple, predictable treatment, while a score of 2 or more in any domain indicates increased complexity and the need for referral. Studies found that DPI scores ≥ 6 were associated with poorer outcomes for root canal retreatment (Tifooni et al. 2019) and teeth were at a higher risk of extraction (Al‐Nuaimi et al. 2020).

The EA is a free web‐based tool which uses several criteria related to tooth anatomy and patient‐related factors (Shah et al. 2018). Each criterion is scored on the level of difficulty (scores: 1 [low], 2 [medium], 5 [high], 9 [extreme]), and criteria scores are then added to provide the overall case score which informs the recommendation regarding the most appropriate treating clinician (Shah and Chong 2018). Low complexity cases (overall scores 1–13) can be managed by general dental practitioners (GDPs); average complexity cases (overall scores 14–17) may require the expertise of either a GDP or a Dentist with Extended Skills (or Special Interest) in Endodontics (DwESEs). High complexity cases (scores 18–25) may require referral to a DwESE or an Endodontic Specialist (SE), and very high levels of difficulty (scores ≥ 26) require referral to a SE (Shah et al. 2020). In a study comparing the educational benefits and user‐friendliness of AAE and EA, both complexity grading systems were found adequate for dental education, but participants preferred EA in terms of user‐friendliness. In addition, EA was shown to be reliable in guiding clinicians to treat or refer to a specialist (Shah et al. 2020).

Introduced in 2021, E‐CAT is an online digital tool that aims to assess the complexity of orthograde root canal treatment cases (www.e‐cat.uk). This tool permits the evaluation of 19 complexity categories and 22 complexity factors, which include patient‐related factors such as complex diagnosis, medical history, history of trauma, psychosocial factors and dental factors. Three complexity classes are defined: Class I (score range of 0 to 5) indicating that the treatment is uncomplicated, Class II (score range of 6 to 11) indicating that the treatment is moderately complicated, and Class III (score > 11) indicating that the treatment is highly complicated (Essam et al. 2021). Inter‐operator validity was shown to be moderate, whereas inter‐operator and intra‐operator reliability were shown to be very good. This may suggest that further improvements to the E‐CAT are needed (Essam et al. 2021).

To the authors' knowledge, no studies have been published comparing EA and E‐CAT for complexity assessment with ECS. Whilst studies are available on DPI, the correlation of DPI scores with other complexity assessment systems has not been established, nor have DPI score thresholds been established to distinguish referrals to DwESEs and SEs. This study aimed to assess and classify the complexity of the cases allocated to the postgraduate programme at Cardiff University Dental Hospital (CUDH) according to ECS, DPI, EA and E‐CAT, and to compare scores for the same case between these four systems.

## 2 Methodology

### 2.1 Ethical Approval

The clinical service evaluation was carried out at the Department of Restorative Dentistry CUDH and was approved by the audit committee and registered on the Audit Management and Tracking (AMaT) platform with health board approval (Dentists/SE/2023‐24/04).

### 2.2 Sample

Sample size calculation was based on a previous study by Long et al. (2022). Two hundred and one case records of consecutive patients were included in this study.

The inclusion criteria consisted of:

– Patients admitted to CUDH and placed on the Master's in Clinical Dentistry in Endodontology waiting list between January 2021 and December 2023.
– Cases with a digital periapical preoperative radiograph stored on the patient archiving and communication system (PACS) used at CUDH (Synapse, Fujifilm Medical Systems U.S.A. Inc., Stamford, Connecticut, USA).

Exclusion criteria consist of:

– Cases without a digital periapical preoperative radiograph saved on PACS.
– Cases with missing data such as Basic Periodontal Examination, medical history, etc.

Patients referred for multiple root canal treatments were included in the study based on the tooth with maximal complexity.

### 2.3 Data Collection

Cases were assessed based on the ECS, DPI, EA and E‐CAT complexity grading systems. The data included in the assessment consisted of the medical history gathered from the patient's records and referral letter, the reason for referral and radiographs that were present on PACS. A standardised method (Schneider 1971) was employed to assess canal curvatures. Additional data was also collected from the patients' records, and information was added anonymously onto a spreadsheet sheet (Microsoft Excel, Microsoft Corporation, Redmond, Washington, USA).

Guidelines were interpreted and areas of ambiguity were discussed and agreed upon (Table 1).

**TABLE 1:** Interpretation of guidelines ambiguity.

Ambiguity	Guideline	Interpretation
Assessment of canal curvature	ECS DPI EA E‐CAT	Canal curvature was assessed by using a standardised method (Schneider 1971)
Root canal anatomy	ECS DPI EA E‐CAT	Maxillary first molars: 4 root canals* Maxillary first premolars: 2 root canals* Mandibular incisors: 1 root canal* Mandibular molars: 3 root canals* *Unless radiograph or dental records suggested otherwise.
Decision on final complexity score	DPI	Teeth that presented full coverage crowns scored a grade of ‘2’ for DPI structural integrity
Root canal negotiability	ECS	If the root canal is obturated to length, the canal was considered as completely negotiable to length unless a separated instrument or a fractured post was present.

Each complexity system differed by its classification name for the complexity levels. ECS referred to three different ‘levels’ of complexity (1, 2 and 3), whereas E‐CAT defined 3 complexity ‘classes’, Class I (0–5, uncomplicated), Class II (6–11, moderately complicated), Class III (> 11, highly complicated). EA referred to 4 ‘levels of difficulty’; low (score of 13), average (score of 14–17), high (score of 18–24), and very high (score of ≥ 25). In order to standardise and compare the classification of complexity between ECS, EA and E‐CAT systems, a common grading system was used which was level ‘1’, ‘2’ and ‘3’, with level ‘1’ being the simplest and level ‘3’ being the most complex. For EA, the ‘high’ and ‘very high’ scores were combined under level 3 complexity. DPI scores for each domain were added to obtain the final score for each case, which were then compared with ECS, EA and E‐CAT to relate the numerical scores of DPI to the different complexity levels of these systems.

Initial examiner calibration was achieved by establishing baseline knowledge of the four complexity grading systems and by evaluating 10 patient records independently. The scores were then compared. In cases where the scores differed, patients' records were reassessed by both examiners and the cases were discussed until agreement was achieved. If agreement between the two examiners could not be attained, patients' records were presented to a third examiner (Consultant in Restorative Dentistry, A.D.) for a final decision.

Twenty patient records were further analysed independently by a postgraduate trainee in Endodontology (N.G.) and a dental core trainee in Restorative Dentistry (H.S.). Inter‐examiner variability was assessed by calculating Cohen's kappa coefficient for each complexity grading system using Microsoft Excel. In the case of disagreement, a patient's records were reassessed by both examiners, and the cases were discussed until agreement was achieved. If agreement between the two examiners could not be attained, the patient's records were presented to a third examiner (Consultant in Restorative Dentistry, A.D.) for a final decision.

Data from the remaining 171 patient records was then assessed and graded by one assessor (N.G.) and reviewed by a consultant in restorative dentistry (A.D.). Sixty patient records were re‐assessed by N.G. and scored again using the four complexity grading systems after an interval of 2 months. Intra‐examiner variability was assessed by calculating Cohen's kappa coefficient for each complexity grading system using Microsoft Excel.

### 2.4 Statistical Analysis

Descriptive statistics and frequency distributions were obtained for ECS, EA, E‐CAT and DPI. The percentage of agreements between ECS, EA and E‐CAT was also calculated. Linearly and quadratically weighted kappa scores and 95% confidence intervals (CI) were calculated to determine the level of inter‐rater agreement (between two independent raters) and intra‐rater agreement (for a single rater) for the ECS, E‐CAT and EA systems. Similarly, linearly and quadratically weighted kappa scores were also calculated to determine the level of agreement between the ECS, E‐CAT and EA systems. As ECS, EA and E‐CAT form ordinal data (allowed values: 1, 2 and 3), non‐parametric measures of correlation (i.e., Spearman's correlation analysis and Kendall's Tau‐b analysis) were calculated between DPI, ECS, EA and E‐CAT. All calculations were performed using SPSS V29.

## 3 Results

A total of 258 case records were assessed. Fifty‐seven were excluded due to missing data, leaving 201 cases for inclusion in this study. This sample comprised 70 incisors (35%), 4 canines (2%), 35 premolars (17%) and 92 Molars (46%). Inter‐examiner and intra‐examiner agreement was statistically significant (*p* < 0.001) and generally high for all complexity grading systems (Table S1).

Most cases were classified as level 3 complexity by ECS (82%, *n* = 165). EA had the highest percentage of level 3 cases (92%, *n* = 184), while E‐CAT had the lowest (74.1%, *n* = 149). Level 2 cases were scored more frequently by E‐CAT (18.4%, *n* = 37) than by ECS (8%, *n* = 16) and EA (7%, *n* = 14). EA had the least level 1 cases (1%, *n* = 2) with similar scoring between ECS (10%, *n* = 20) and E‐CAT (7.5%, *n* = 15).

**FIGURE 1:**
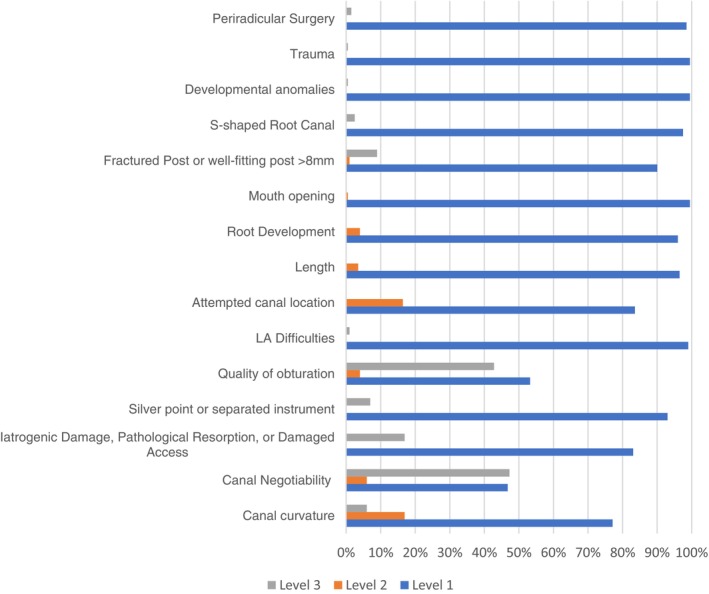
Complexity levels identified when using ECS complexity system for each patient/treatment factor in percentage (*n* = 201).

**FIGURE 2:**
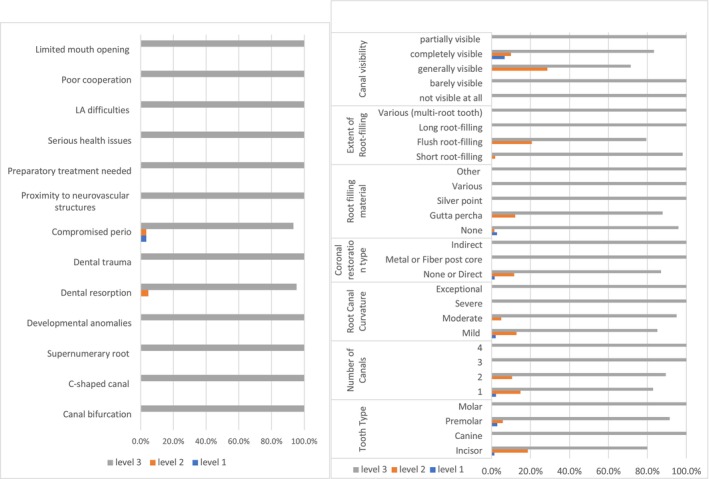
Complexity levels identified when using EA complexity system for each patient/treatment factor.

**FIGURE 3:**
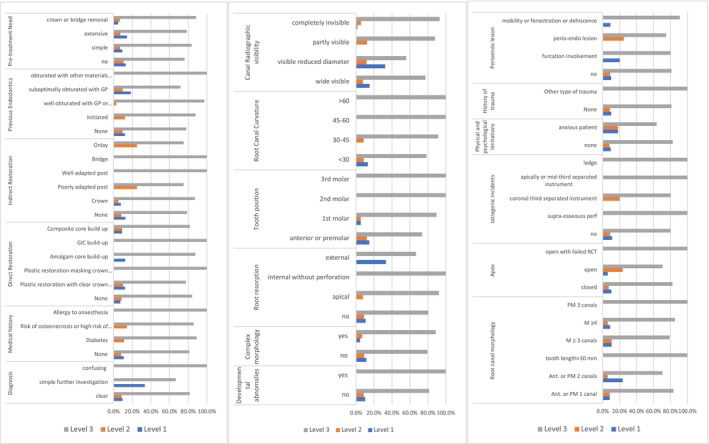
Complexity levels identified when using E‐CAT complexity system for each patient/treatment factor.

For ECS, ‘canal negotiability’ was the most frequent complicating factor, leading to a complexity score of 3 (47%, *n* = 95) (Figure 1). The ambiguity within the algorithms of EA and E‐CAT prevented the identification of weighting given to factors that contributed to the complexity of the endodontic treatment when using EA and E‐CAT (Figures 2 and 3).

The scores for ECS and E‐CAT coincided perfectly in 78.6% of the cases, with E‐CAT scoring higher in 5 cases and substantial levels of agreement between the two (*κ* = 0.647, 95% CI: 0.517 to 0.776, *p* < 0.001) (Table 2). For ECS and EA, the scores coincided perfectly in 79.6% of the cases, with EA scoring higher in 18 cases and fair levels of agreement between these tools (*κ* = 0.290, 95% CI: 0.113 to 0.466, *p* < 0.001). For E‐CAT and EA, the scores overlapped in 77.6% of the cases, with EA scoring higher in 13 cases and fair levels of agreement (*κ* = 0.385, 95% CI: 0.226 to 0.544, *p* < 0.001) (Table 2).

**TABLE 2:** Linearly and quadratically weight kappa statistics (*p* < 0.001 in all cases).

Complexity grading systems	Linear weights (95% CI)	Quadratic weights (95% CI)
E‐CAT vs. ECS	0.536 (0.406 to 0.667)	0.647 (0.517 to 0.776)
E‐CAT vs. EA	0.324 (0.191 to 0.456)	0.385 (0.226 to 0.544)
ECS vs. EA	0.226 (0.078 to 0.375)	0.290 (0.113 to 0.466)

**FIGURE 4:**
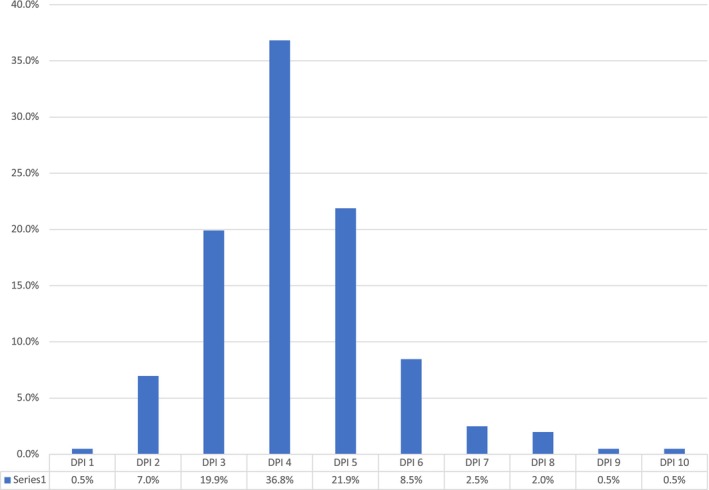
Percentage of different DPI scores (*n* = 201).

**FIGURE 5:**
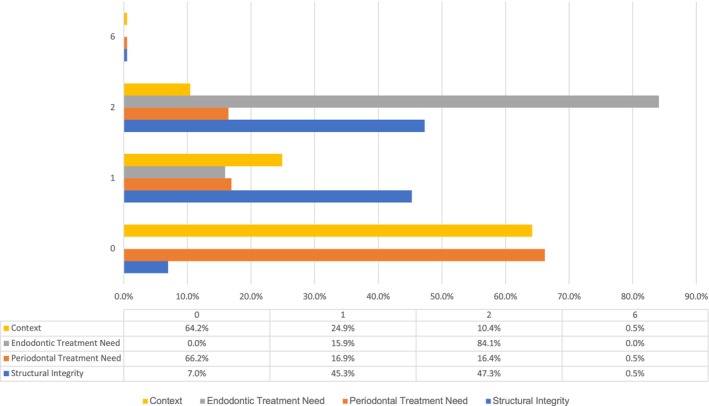
Complexity levels identified when using DPI complexity system for each patient/treatment factor (*n* = 201).

**FIGURE 6:**
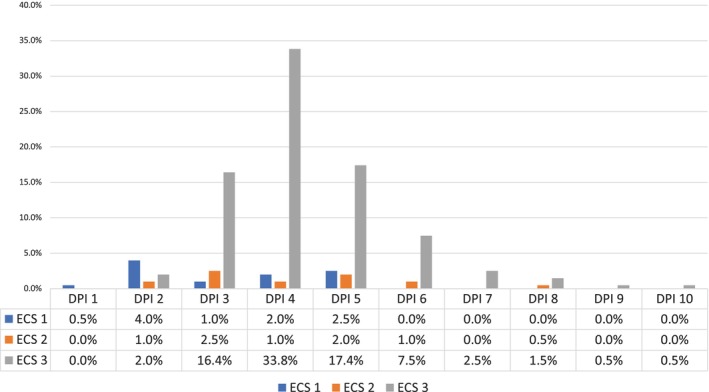
Chart representing combined DPI‐ECS scores in percentage (*n* = 201).

**FIGURE 7:**
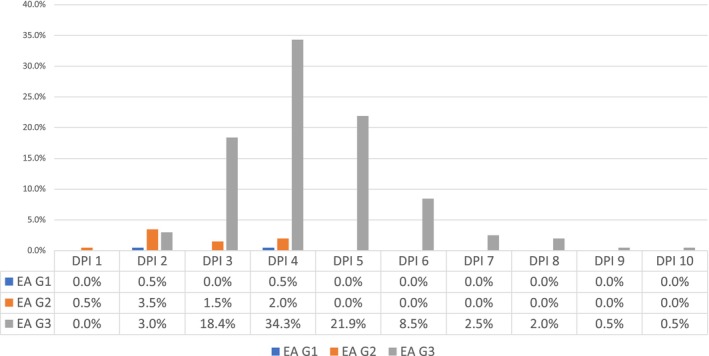
Chart representing combined DPI‐EA scores in percentage (*n* = 201).

**FIGURE 8:**
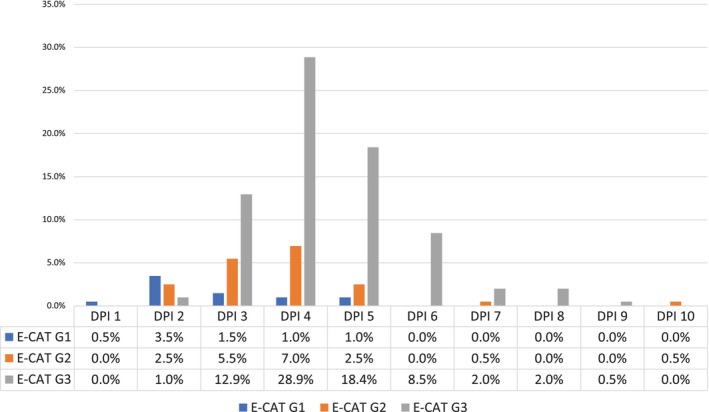
Chart representing combined DPI–E‐CAT scores in percentage.

DPI scores ranged from 1 to 10 (mean = 4, 95% CI = 4.05–4.43) with score 4 being the most frequent (36.8%, *n* = 74) (Figure 4). The factor ‘endodontic treatment need’ was the most frequent complicating issue (84.1% cases, *n* = 169) (Figure 5). When comparing DPI with ECS, EA and E‐CAT, cases that were classified as level 3 with the latter systems corresponded most frequently with DPI score 4 (score range 2–10), but with more variability for other complexity levels (Figures 6, 7, 8).

**FIGURE 9:**
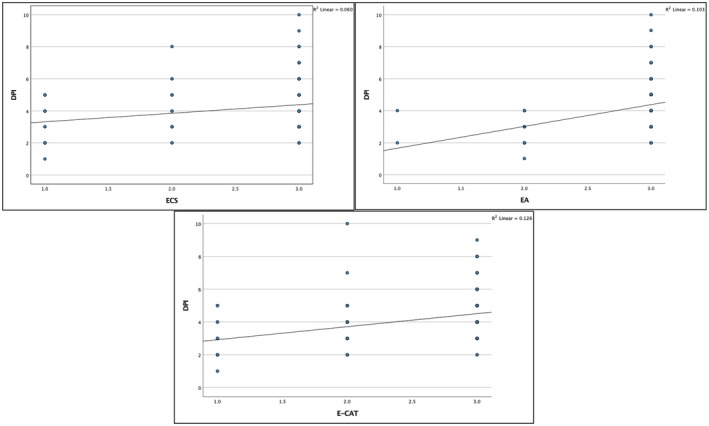
Scatter plot illustration of the correlation between DPI and ECS (a), EA (b) and E‐CAT (c); there is a statistically significant but weak positive correlation (*p* < 0.05).

Spearman's correlation analysis and Kendall Tau‐b analysis showed a statistically significant but weak positive correlation between DPI and ECS, EA and E‐CAT (Table 3 and Figure 9).

**TABLE 3:** Spearman's Correlation analysis and Kendall Tau‐b analysis investigating the association between DPI and ECS, EA and E‐CAT.

Complexity grading system	Spearman's correlation coefficient (*r*_s_)	*p* (*r*_s_)	Kendall Tau‐b Correlation Coefficient (*r*_k_)	*p* (*r*_k_)
ECS	0.202	0.004	0.179	0.004
EA	0.344	< 0.001	0.308	< 0.001
E‐CAT	0.364	< 0.001	0.324	< 0.001

## 4 Discussion

In the last two decades, endodontic complexity assessment has developed from a tooth‐centered approach into a patient‐oriented approach (Shah et al. 2018; Tifooni et al. 2019; Essam et al. 2021). In this study, ECS, E‐CAT and EA tools found that most endodontic referrals received were level 3 complexity, and DPI scores of 3–5 accounted for 78.1% of the referred cases.

Both E‐CAT and EA included detailed parameters as compared with ECS, which suggested a more clinically relevant approach to complexity assessment. An example is ‘radiographic canal visibility,’ which was further objectively sub‐classified by EA and E‐CAT, whereas ECS relied on a more subjective interpretation of canal negotiability (Kuyk and Walton 1990; Pesonen et al. 2021). More difficult cases with extensive canal calcification can therefore be allocated to adequately skilled clinicians who use modern armamentarium (magnification, illumination, ultrasonics, cone‐beam computed scanning and guided access).

Further, E‐CAT demonstrated a more nuanced approach to complexity scoring compared with ECS and EA owing to its more detailed scoring criteria for each factor. For example, E‐CAT distinguished between clinically visible and non‐visible separated instruments, aligning with studies that reported lower retrieval rates for non‐visible instruments (Nevares et al. 2012). Similarly, E‐CAT considered the location of perforations (sub‐osseous vs. supra‐osseous), which can impact treatment complexity (Estrela et al. 2018).

Whilst canal curvatures were assessed by all complexity grading systems, the definitions of severity varied between systems and contributed to differences in complexity scores, with EA assigning higher scores for similar curvatures. With the development of modern heat‐treated martensitic instruments which enable more predictable shaping, the variable latitude of canal curvature definitions needs international consensus. Modern complexity assessment tools also beneficially recognise concomitant patient factors such as limited mouth opening and posterior tooth position which can influence the overall operative difficulty and treatment risk during the management of curved canals.

In the current study, an attempt has been made to validate the DPI with a nationally recognised standard (ECS) in the United Kingdom, as well as the EA and E‐CAT. A weak positive correlation was observed between DPI scores and the other complexity grading systems. DPI overall scores 1–2 generally correlated with level 1 complexity in ECS and E‐CAT but EA tended to assign level 2 complexity scores within this DPI range. This can be explained by cumulatively low scores (0 or 1) accrued through different domains in DPI for a small cohort of patients (4%–6%) who had a DPI score of 2 but were classed as either level 1 or 2 by other systems. Additionally, the current study is based in a dental hospital where the referred cases are more complex, with only very few patients scored as complexity grade 1 across the other grading systems. The paucity of data for level 1 and 2 cases coupled with the weak correlation between the complexity systems made it difficult to validate a comparable cut‐off score for DPI through this study, which is a limitation of this project. A future study can be performed in the primary dental care sector to help gauge suitable cut‐off scores for level 1 and 2 cases.

An overall cut‐off score of DPI 3 equated with complexity grading 3 in other systems in this study. This can be explained by the cumulative nature of scoring in DPI domains. Whilst a level 2 score in the endodontic domain of the DPI would warrant an advanced endodontic care pathway, the current study showed that such cases had an overall score > 2 because additive points were gained in other domains concurrently, such as the need for an indirect restoration, the patient's general health and the wider context of the patient's dentition. Therefore, for DPI scores ≥ 3, there was more consistency between DPI and the remainder complexity grading systems, which indicated that referral was warranted to a specialist unit.

Assessment of the structural integrity of the tooth in DPI is presented as a limitation in the current study because only radiographic evaluation was performed. Accurately evaluating the integrity of tooth structure, particularly beneath full coverage crowns, remains a challenge without dismantling the restoration (Abbott 2004), potentially leading to inaccurate pre‐operative complexity scores.

The results of this clinical service evaluation highlight the need for a standardised approach to case complexity assessment. While all the evaluated complexity grading systems demonstrated good inter‐examiner and intra‐examiner reliability, the evolution of case complexity assessment into endodontic risk assessment will facilitate patient management by the appropriate clinicians and seek to offer patients informed choices about treatment.

## 5 Conclusions

Within the limitations of this clinical service evaluation, it was possible to conclude that the majority of cases treated at CUDH were of high complexity (level 3) using ECS, E‐CAT and EA. This was appropriate for secondary care settings. E‐CAT assigned slightly lower complexity scores compared with ECS and EA, potentially due to the more detailed approach to factors like instrument location, perforation type and a wider range of curvature classification. Scores of 3 to 5 of DPI accounted for the majority of referred cases to CUDH. A weak positive correlation was found between DPI and the other three complexity grading systems (ECS, E‐CAT and EA). DPI's broader assessment justifies the current cut‐off of score 3 for specialist referral due to the increased agreement with ECS, E‐CAT and EA at this threshold.

## Author Contributions

N.G. involved in methodology, including data collection, investigation, statistical analysis and writing of the original draft. H.S. involved in data collection. E.L. and J.H. involved in reviewing and editing of the draft. D.F. involved in the statistical analysis. A.D. involved in conceptualization, methodology, investigation, reviewing and editing of the draft.

## Ethics Statement

This service evaluation was approved by the audit committee and registered on the Audit Management and Tracking (AMaT) platform with health board approval (Dentists/SE/2023‐24/04).

## Conflicts of Interest

The authors declare no conflicts of interest.

## Supporting information


**Table S1:** Inter‐examiner & intra‐examiner reliability: Quadratically‐weighted kappa statistics (95% confidence intervals) showing statistical significance (*p* < 0.001).

## Data Availability

The data that support the findings of this study are available from the corresponding author upon reasonable request.
