# Supplementary material for: Assessment of Case Complexity of Root Canal Treatments Using Contemporary Complexity Grading Systems: A Clinical Service Evaluation
Source: Int Endod J. 2025 Oct 6;59(1):47–56. doi: 10.1111/iej.70039 (PMC12701745; doi:10.1111/iej.70039)
Supplement: Supplementary file 1 — Table S1: Inter‐examiner & intra‐examiner reliability: Quadratically‐weighted kappa statistics (95% confidence intervals) showing statistical significance (p < 0.001). [file IEJ-59-47-s001.docx]

Supplementary Table 1- *Inter-examiner & Intra-examiner reliability: Quadratically-weighted kappa statistics (95% confidence intervals) showing statistical significance (P<0.001)*

|  | Inter-examiner reliability | Intra-examiner reliability |
| --- | --- | --- |
| ECS | 0.464 (-0.149-1.078) | 0.818 (-.537-1.098) |
| DPI | 0.778 (0.451-1.1.04) | 0.98 (0.96-1) |
| E-CAT | 0.903 (0.754-1.052) | 0.967 (0.899-1.034) |
| EA (common-grading system: 3 levels) | 1 (1-1) | 1 (1-1) |
| EA (with segregation of high & very high complexity) | 0.755 (0.489-1.02) | 0.842 (0.667-1.007) |
